# Progressive Study on the Non-thermal Effects of Magnetic Field Therapy in Oncology

**DOI:** 10.3389/fonc.2021.638146

**Published:** 2021-03-17

**Authors:** Aoshu Xu, Qian Wang, Xin Lv, Tingting Lin

**Affiliations:** ^1^College of Instrumentation and Electrical Engineering, Jilin University, Changchun, China; ^2^Key Laboratory of Geophysics Exploration Equipment, Ministry of Education of China, Changchun, China

**Keywords:** magnetic fields, anti-tumor, molecular mechanism, static magnetic fields, low-frequency magnetic fields

## Abstract

Cancer is one of the most common causes of death worldwide. Although the existing therapies have made great progress and significantly improved the prognosis of patients, it is undeniable that these treatment measures still cause some serious side effects. In this context, a new treatment method is needed to address these shortcomings. In recent years, the magnetic fields have been proposed as a novel treatment method with the advantages of less side effects, high efficiency, wide applications, and low costs without forming scars. Previous studies reported that static magnetic fields (SMFs) and low-frequency magnetic fields (LF-MFs, frequency below 300 Hz) exert anti-tumor function, independent of thermal effects. Magnetic fields (MFs) could inhibit cell growth and proliferation; induce cell cycle arrest, apoptosis, autophagy, and differentiation; regulate the immune system; and suppress angiogenesis and metastasis *via* various signaling pathways. In addition, they are effective in combination therapies: MFs not only promote the absorption of chemotherapy drugs by producing small holes on the surface of cell membrane but also enhance the inhibitory effects by regulating apoptosis and cell cycle related proteins. At present, MFs can be used as drug delivery systems to target magnetic nanoparticles (MNPs) to tumors. This review aims to summarize and analyze the current knowledge of the pre-clinical studies of anti-tumor effects and their underlying mechanisms and discuss the prospects of the application of MF therapy in cancer prevention and treatment.

## Introduction

Cancer is a serious threat to human health and one of the leading causes of death worldwide. According to estimates with regard to morbidity and mortality for 36 kinds of cancers in 185 countries, about 18.1 million new cancer cases plus 9.6 million cancer-associated deaths happened in 2018 (1). Among these cancers, the highest incidence types are lung (11.6%), breast (11.6%), prostate (7.1%), and colorectal (6.1%) cancers. At present, the primary options for advanced cancer treatments, namely chemotherapy and radiotherapy, always have some limitations such as severe side effects and drug resistance (2–4). It is necessary to develop new therapies to address these disadvantages. In this context, more attention was paid for alternative treatments involving some non-invasive approaches like light, heat, electrical field, magnetic field (MFs), and ultrasound therapies (5–9), which are of high efficiency and incur low costs without inducing infections or forming scars. Among them, the MF therapy has been studied a lot in recent years, as early as 1971, when Weber et al. (10) validated the inhibitory effects of MFs on tumor-bearing mice. Over the next few decades, many researchers have explored this phenomenon and put forward more evidence about the relevant mechanisms (11–13); at the same time, clinical trials demonstrated its advantage in relieving clinical symptoms, and improving the quality of life of patients with recurrent and rapidly progressing tumors (Table 1) (17). Early studies have shown that in the field of cancer treatment, MFs have potential application prospects with few side effects and wide applications. MFs could non-invasively induce the death of cancer cells, whereas lymphocytes showed little necrosis *in vitro* (18, 19). In other medical studies, the MF therapy has been reported to have beneficial results in peripheral nerve regeneration (20), osteo-necrosis (21), and injury-induced osteoporosis (22). MFs at frequencies above 100 kHz predominately show thermal effects; otherwise, they would exert non-thermal effects (23). Recently, non-thermal biological effects of MFs have been reported in many aspects, among which are studies on tumor treatment. The inhibitory effects of static magnetic fields (SMFs) and low-frequency magnetic fields (LF-MFs, with frequency below 300 Hz) have been studied against a wide variety of human cancer cell lines, such as leukemia (24–31), fibrosarcoma (32), colon carcinoma (32–34), and breast cancer (35–40). Furthermore, MFs suppress the growth of Lewis lung carcinoma (LLC) (41) and Ehrlich ascites carcinoma (42, 43) *in vivo*, and even prolong survival and improve the general symptoms of 21 patients with advanced gastric cancer (44). MFs have shown to exert anti-tumor action through various pathways and multiple molecular mechanisms, such as the inhibition of cell growth and proliferation; the induction of apoptosis, cell cycle arrest, and autophagy; participation in immune regulation as well as depression of angiogenesis, and metastasis; and promotion of differentiation. Of interest, they are effective in combination therapies with chemotherapeutic agents and magnetic nanoparticles (MNPs).

**Table 1:** Early research foundation of magnetic fields (MFs) in tumor suppression.

**Year**	**Some important breakthroughs**	**Reference**
1961	Mulay et al. discovered tumor cells exposed to MFs showed complete degeneration.	(14)
1971	Weber et al. confirmed that the non-homogeneous MF consistently prolonged the life spans and slowed down the growth of tumors in mice.	(10)
1971–1975	Mizushima and Degen et al. reported the anti-inflammatory effects of MFs.	(11, 15)
1999	Chakkalakal et al. found that the MFs had the potential to promote the effects of chemotherapeutic drugs and reduced the dosage and side effects.	(16)
2001	Tofani et al. demonstrated that static plus low-frequency magnetic fields (LF_MFs) induced the apoptosis of tumor cells.	(13)
	Douglas et. al. described the inhibitory effects of MFs on angiogenesis during tumor growth.	(12)
2010	Vasishta found MFs alleviated the clinical symptoms and improved the quality of the life of patients with anaplastic astrocytoma.	(17)

## Aim and Searching Criteria

### Thermal Effects and Non-thermal Effects by (MF) Therapy

Two conditions of the molecular mechanism, namely thermal effects and non-thermal effects, are involved in MF-induced biological effects (23). According to IEEE C95.1-2019, thermal effects are defined as “changes associated with heating of the whole body or an affected region sufficient to induce a biological effect.” Electro-stimulation is the dominant effect at low frequencies and thermal effects dominate above radio frequencies. The International Commission on Non-Ionizing Radiation Protection (ICNIRP) gives a more detailed description of electro-magnetic fields at radio frequency (100 kHz−300 GHz), which could penetrate the body and cause a vibration of charged or polar molecules inside, resulting in friction and heat. Thermal effects lead to an increase in bulk temperature, which would thermally induce membrane depolarization, excitation, and breakdown and show a distinct side effect on the organisms (45). Non-thermal effects could be described as direct interactions of MF with biological cells that are not associated with any heating but are associated mainly with electro-stimulation (23, 46). Based on the physical mechanisms, extremely LF-MFs (<300 Hz) are regarded as non-thermal effects (47).

### Aim and Scope

Magnetic fields are generally generated by permanent magnets or electric currents, and there are several classification methods for MFs. According to the mechanism of the generation of MFs, they are divided into permanent magnetic fields and electromagnetic fields. While the variation rate of the intensity of the MF with spatial displacement is equal to 0, it is a uniform MF; otherwise, it is a gradient magnetic field (GMF). Moreover, if the distribution of the MF changes with time, they are classified as SMFs and non-SMFs, such as alternating magnetic fields (AMFs), pulsed magnetic fields (PMFs), and rotating magnetic fields (RMFs). In consideration of their working frequency, the MF is classified into low frequency (LF) (<300 kHz), medium frequency (MF) (300 kHz−3 MHz), and high frequency (HF) (>3 MHz). According to the Regulations (2012) of the International Telecommunication Union (ITU), LF-MFs are further divided into tremendously LF(<3 Hz), extremely LF (3–30 Hz), super LF (30–300 Hz), ultra LF (300–3000 Hz), very LF (3–30 kHz), and LF (30–300 kHz) (48). Many studies have shown that SMFs and LF-MFs (f <300 Hz) exerted anti-tumor effects, in which the temperature was maintained at around 37°C for cell culture *in vitro* and excluded thermal effects (30, 49, 50). To comprehend the non-thermal effects of the MF therapy on cancers, we focus on the abovementioned MF types in this review, aiming at describing the state of the art of MF therapy, discussing the current understanding of the underlying anti-cancer mechanisms, and outlining future therapeutic perspectives in oncology. Common setups, types, exposure direction, and duration of the action are summarized in Table 2.

**Table 2:** Common setups, types, exposure direction, and duration of the action of MFs used in anti-tumor studies.

**Characteristics**	**Terminology**	**Graphic representation**	**Description**	**Reference**
Common MF setups	Permanent magnet	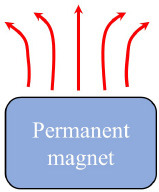	One permanent magnet	(51)
	Permanent magnets	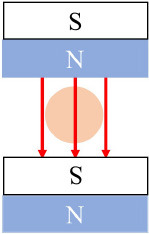	Two permanent magnets aligned in the same direction	(52)
	Solenoid coils	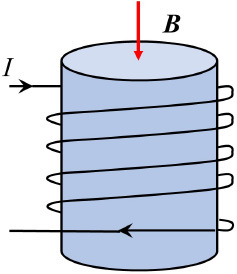	***B*** = *u*_0_ × (N/L) × *I* N - turn ratio of coils; L - solenoid length	(27, 53)
	Uniform (Helmholtz geometry)	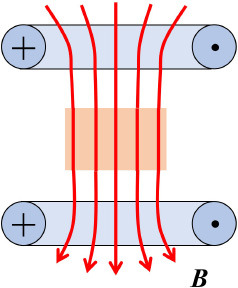	***B***(x, y, z) ≈ const grad ***B*** ≈ 0	(54)
MF types	Static magnetic fields (SMFs)	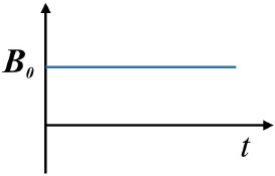	***B = B***_0_ = const	(24, 51)
	Alternating magnetic fields (AMFs)	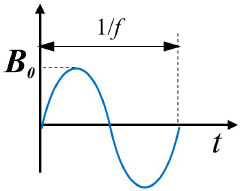	***B*** = ***B***_**0**_ × sin(2π*ft*) *f*- field change frequency;	(39)
	Pulsed-magnetic fields (PMFs)	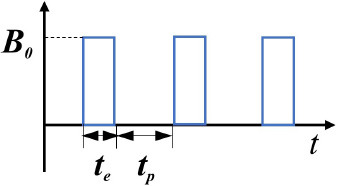	***t**_***e***_***-** field action duration ***t**_***p***_***-** pause duration	(35)
	Gradient magnetic fields (GMFs)	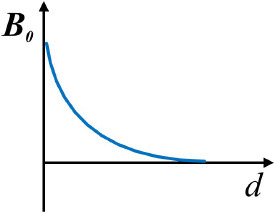	***B*** is proportional to 1/d^2^ d - the distance away from the magnets	(55)
Orientational	Parallel	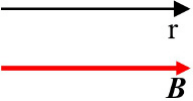	***B*** parallel to ***r*** **r -** the plane of cell culture dish	(26)
	Vertical	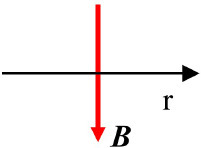	***B*** perpendicular to **r**	(33)
	Rotating	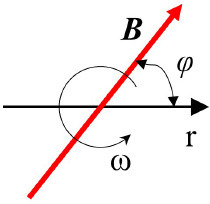	***B*** **=** const φ = ω**t**	(52)
	Random	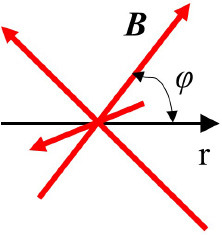	***B*** ~ variable φ ~ variable	(40, 56)
Exposure	Continuous	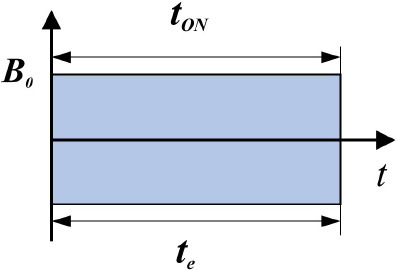	***t*_*ON*_ = *t*_e_** ***t***_***ON***_ - field action duration; ***t***_***e***_-exposure duration	(57)
	Intermittent	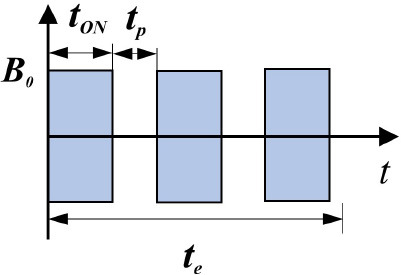	***t***_***ON***_**-** pause duration ∑**t**_***ON***_<***t***_***e***_	(35)

In this review, we focus on the non-thermal effects of SMFs and LF-MFs (<300 Hz) on cancer cells and their applications in cancer treatment. The review aims to highlight the critical areas regarding the uses of MF therapy, which are not fully understood and need to be investigated further.

### Searching Criteria

The literature search was carried out with Scopus, Google Scholar, PubMed, Web of Sciences (ISI Web of Knowledge), Medline, and Wiley Online Library databases. Available publications (in English) in peer-reviewed journals on the biological effects of SMFs and LF-MFs between 2008 and 2019 were selected for analysis. We focus on SMF- and LF-MF-induced anti-tumor effects in *in vivo* and *in vitro* studies. The studies on the influence of SMFs and LF-MFs on other organs and systems were excluded from the literature. The keywords used for the literature research were “apoptosis,” “cell cycle arrest,” “autophagy,” “angiogenesis,” “immune,” “inflammation,” “differentiation” (as a combination with “low frequency electromagnetic fields” or “static magnetic fields,” and “tumor” or “cancer” or “oncology”).

## The Effects of MFs on Cell Proliferation, Cell Cycle Arrest, Cell Apoptosis, and Autophagy

Magnetic fields exert their function through various pathways and multiple targets. A large number of recent studies have shown that MFs have anti-tumor effects by inhibiting cell proliferation and inducing cell cycle arrest, apoptosis, and autophagy.

### Cell Cycle Arrest

The cell cycle, which consists of the G1, S, G2, and M phases, is a very complex and delicate regulation process closely related to cell differentiation, growth, and death. Abnormal expressions of some cell cycle proteins could cause uncontrolled replication of cancer cells; so it is a promising therapy to target cyclins (58).

**Figure 1:**
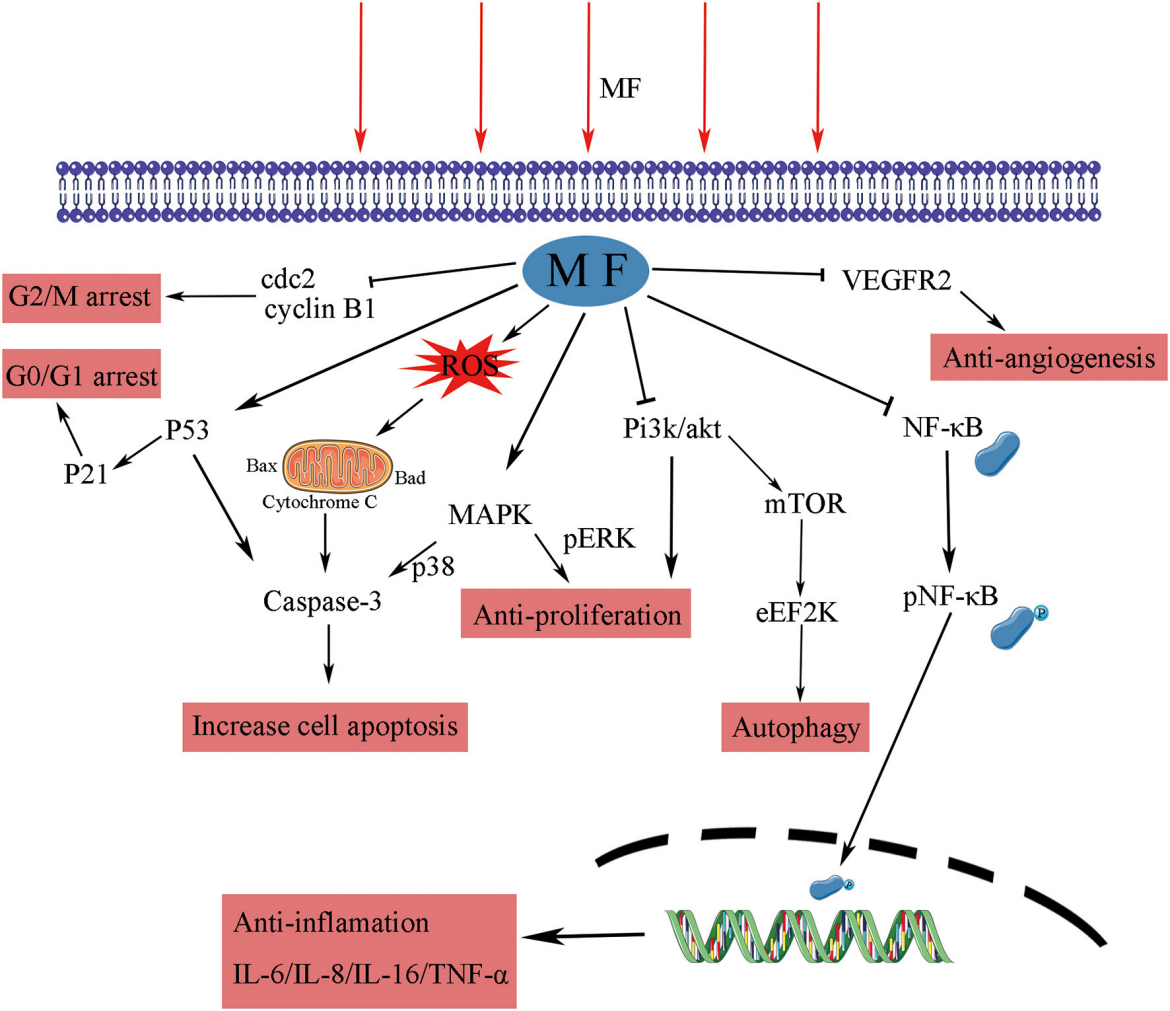
The effect of MFs on different signaling pathways and their molecular targets. MF, magnetic field; cdc, cell division control protein kinase; ERK, extracellular signal-regulated kinase; MAPK, mitogen-activated protein kinase; Akt, protein kinase B; Pi3k, phosphatidylinositol 3-kinase; mTOR, mechanistic target of rapamycin kinase; eEF2K, eukaryotic elongation factor 2 kinase; NF-κB, nuclear factor kappa B; IL, interleukin; TNF, tumor necrosis factor; VEGFR, vascular endothelial growth factor receptor.

DNA integrity is critical to cells; common radiotherapy and most of the chemotherapies exert their function by damaging the cancer cells of DNA, which would inhibit proliferation at cell cycle checkpoints and lead to cell death (59). SMFs (8.8 mT, 12 h) enhanced the killing potency of cisplatin, adriamycin, and paclitaxel by triggering DNA damage, inducing cell ultrastructure alteration, and arresting K562 cells at the G2/M phase (27–29). RMFs (0.4 T, 7.5 Hz, 2 h/day) inhibited the growth of B16-F10 *in vitro*, elevated the survival rate, and inhibited the proliferation in the lung metastasis model mice, where an increase in the G2/M phase was detected (52). CDK1-cyclin B, also known as cell division control protein kinase 2-cyclin B (cdc2-cyclin B) functions at the G2/M phase of the cell cycle, to accelerate cell mitosis (60). SMFs (200 ± 60 mT, 48 ± 4 h) induced human malignant glioblastomata, such as U87 and U251, to arrest the G2/M phase by downregulating the expressions of cyclin B1 and CDK1 (61). The p53 protein is a critical participant in the signal transduction pathway which mediated apoptosis and G1 cell cycle arrest in mammalian cells (62). LF-MFs significantly inhibited tumor growth, induced cell senescence, inhibited iron metabolism of the LLC murine model, and the *in vitro* induced G0/G1 phase arrest of A549 lung cancer cells *via* stabilizing p53 protein and activation of the P53-miR-34a-E2F1/E2F3 pathway (41). In addition, earlier experiments with high risk BE(2)-C neuroblastomas continuously exposed in 50 Hz, 1mT LF-MF for 72 h led to an enhanced cell response to ATRA, along with an increase in the levels of p21, Cdk-5, and G0/G1 population (63). A 24-h exposure of 50 Hz, 100 uT LF-MF exposure slowed down the progression of the cell cycle, which is associated with the regulation of p21 in early response (64). These data indicate that MFs are found to arrest cells at different stages, thus leading to anti-proliferation effects on cells by modulating cell cycle regulatory proteins, as summarized in Figure 1.

### Apoptosis

Apoptosis, which is a form of programmed cell death as well as a target for anti-tumor therapies, plays an important role in cancer treatment (65). There are two main apoptosis pathways: one occurs through the mitochondrial pathway (intrinsic pathway) and another through the cell death receptor pathway (extrinsic pathway). The intracellular mitochondrial pathway is mainly regulated by B-cell lymphoma-2 family, which could promote the formation of channels in the extracellular membrane of mitochondria to change the permeability, release a variety of apoptosis-related proteins to activate caspase, and induce apoptosis (66, 67). Targeting some pro-apoptosis proteins, anti-apoptosis proteins, and mitochondrial membrane permeability are attractive for cancer therapy, by contributing to the occurrence of the intrinsic apoptosis pathway (68, 69).

Magnetic fields have been shown to induce apoptosis in human tumor cells studied *in vitro*. A 50-Hz LF-MF (5.1 mT, 2 h/day) inhibited proliferation of nephroblastoma and neuroblastoma cells, induced apoptosis *in vitro*, and promoted the efficacy of cisplatin *in vivo* (49). Reactive oxygen species (ROS) and mitochondria play an important role in the induction of apoptosis (70), and an increase in ROS levels can lead to cytochrome c release and mitochondrial apoptosis (54). The MF treatment has been shown to promote the generation of ROS in many studies (31, 71, 72), with exposure within a 60 Hz sinusoidal MF for 48 h in induced human prostate cancer for DU145, PC3, and LNCaP apoptoses, associated with the accumulation of ROS in an intensity-dependent manner (73). Generally, apoptosis provoked by genotoxins is largely due to DNA damage (74), while DNA double-strand breaks (DSBs) are one of the most severe types of DNA lesions (75). Repetitive exposure to LF-MFs induced DNA damage and accumulation of DSBs and triggered apoptosis in Hela and MCF7 cell lines (35, 76). As p53 is a tumor suppressor gene that plays a pivotal role in apoptosis, PMFs could trigger apoptosis cell death by upregulating the p53 level and through the mitochondrial-dependent pathway (57). LF-MFs (300 mT, 6 Hz, 24 h) also induced apoptosis by suppressing protein kinase B (Akt) signaling, activating p38 mitogen-activated protein kinase (MAPK) signaling, and caspase-9, which is the executor of the mitochondrial apoptosis pathway (77).

The findings of these studies have shown that MFs affect apoptosis in the cancer cell lines of various origins. However, at present, there are few studies in this area, and further studies are required for detailed mechanisms. The proposed mechanism involved in the effects of MFs on tumor cell apoptosis is shown in Figure 1.

### Autophagy

Autophagy is thought to have a therapeutic potential to prevent cancer development, but whether to enhancing or inhibiting it will achieve the desired anti-tumor effects remains questionable (78). Autophagy could be ascertained by detecting LC3-II, a marker of autophagic vesicle accumulation (79). To date, miRNAs were proved to involve in the modulation of a wide range of biological processes, including apoptosis and autophagy (80). The expression of the autophagy marker, LC3-II, detected by Western blotting and GFP-LC3 puncta-formation assay examined by confocal microscopy, showed that RMFs (0.4T, 7.5 Hz, 4 h/day) induced autophagic cell death and suppressed cancer growth *in vitro* and *in vivo*. The main mechanism involved the upregulation of the expression level of miR-486, which was targeting BCAP, the inhibition of Akt/mechanistic target of rapamycin kinase (mTOR), and the induction of autophagy by RMF (81). These findings showed the potential of MF in triggering the autophagic cell death.

## The Effects of MFs on the Immune System

The immune function in an organism exerts an essential role in the occurrence and metastasis of tumors. The RMF (0.4T, 7.5 Hz, 2 h/day) has the capacity to elevate the survival rate of tumor-bearing by modulating the immune response and functions of innate immune cells and adaptive immune cells, such as regulating cytokine production in mice serum, promoting T-cell polarization in the spleen, preventing the differentiation of the regulatory cells (Tregs), and increasing the expression of CD40 in dendritic cells (52). Furthermore, analogous results were discovered in mouse H22 hepatocellular carcinoma, with an enhanced anti-tumor immune response; the inhibition of tumor growth; and the suppression of interleukin-6 (IL-6), granulocyte colony-stimulating factor (G-CSF), and keratinocyte-derived chemokine (KC). Meanwhile, the MF exposure was associated with the activation of macrophages and dendritic cells, enhancement of the profiles of CD4+T and CD8+T lymphocytes, the balance of Th17/Treg, and the reduction of the inhibitory function of Treg cells *in vivo* (82). A combination of SMF with AMF stimulated the production of tumor necrosis factor-α (TNF-α), interferon-gamma, IL-2, and IL-3 in healthy mouse cells, inhibited solid tumor growth, and enhanced the average lifespan, after daily exposure for 2 h within 14 days (83).

Inflammation is a key factor in the immune response to injury and infection; some studies have shown that the progression of various cancers may be closely related to chronic inflammation (84, 85). Exposure to PMF with an intensity of 40 Gauss and frequency below 30 Hz for 48 h decreased the production of the inflammation marker TNF-α and the transcription factor nuclear factor kappa B (NF-κB). In RAW 264.7 macrophage-like cells, induced by LPS, this regulation process could be appropriately applied to patients with sepsis (86). The upregulation of A2A and A3ARs adenosine receptor mRNA levels by the PMF (1.5 ± 0.2 mT, 75 Hz, 24 h) mediated the anti-inflammation effect, induced the decrease of NF-κB expression, upregulated p53, and induced apoptosis in tumor cells (57). The GMF (6.39–513.69 mT, 24 h) significantly inhibited the release of pro-inflammatory cytokines, IL-6, IL-8, and TNF-α, from macrophages and assisted the production of anti-inflammatory cytokine, IL-10, when treate alone for 24 h and then combined with LPS (87). A similar response was induced by the PMF in N9 microglial cells (88). An *in vitro* study found that SMF (0.4 T, 6 h) could attenuate LPS-induced neuro-inflammatory responses in BV-2 cells, and this effect was associated with increased microglial membrane rigidity and downregulation of IL-6 release (89). MFs have the ability to enhance the immune response of the body to tumors by modulating the functions of immune cells and inhibiting chronic inflammation (Figure 1); while the regulation of the immune system is complex, further research is needed to explain the relationship.

## The Effects of MFs on Angiogenesis, Metastasis, and Differentiation

### Angiogenesis

Angiogenesis is a critical physiological and pathological process in embryo development, tumor development, and metastasis. The formation of new blood vessels gradually has become an essential therapeutic target in cancer treatment, ischemic diseases, and chronic inflammation (90). Vascular endothelial cell migration is an important part of the angiogenesis process of tumors, and vascular endothelial growth factor (VEGF-A, VEGF) and its receptor-2 (VEGFR-2) play an important role in tumor angiogenesis, which gradually becomes a target in anti-tumor therapy (91). The SMF (600 mT, 10 days) has been shown to inhibit angiogenesis by reducing vessel diameters, the functional vessel density (FVD), and red blood cell velocity to retard vessel maturation by *in vivo* tests (92). After 24-h exposure in the GMF (0.2–0.4 T, 2.09 T/m), the proliferation ability of human umbilical vein endothelial cells (HUVECs) was significantly inhibited. In the chick embryo chorioallantoic membrane (CAM) model, vascular numbers of continuously exposure treatment group (7–11 days) are fewer than those in the control group, which is consistent with the results in matrigel plugs models (55). Sinusoidal MF (1 mT, 50 Hz,72 h) inhibited the formation of tubule-like structures and downregulated the process and migration of HUVECs by reducing the expression and activation levels of VEGFR2 (93). A combination therapy of MF (0.04 T,50 Hz, 1 h) and saffron had synergic effects on VEGFR2 gene expression; they reduced the VEGFR-2 level by 36%, while MF alone only induced a 20% decline in human breast cancer cells (37). A therapeutic MF device, which could generate a defined 120 Hz semi sine wave signal with variable intensity (10–20 mT), was tested for the optimal intensity and treatment period of MF therapy for breast cancer. Exposure to 20 mT for 10 min two times a day within 12 days was the most effective tumor suppressor; the MF treatment reduced the vascular (CD31 immuno-histochemically positive) volume fraction (94). These studies indicate that the MF theraphy is a promising therapy that may target tumor angiogenesis through the pathways showed in Figure 1.

### Metastasis

Tumor metastasis is the leading cause for death in patients with cancer, and up to 90% of cancer deaths occur due to metastasis. After intermittent treatment for several weeks, a therapeutic electromagnetic field (15 mT, 10 min/day) has proved to inhibit the metastatic spread in the nude mice injected with breast cancer cells, which might be associated with the decrease in volume density of blood vessels (95). Furthermore, the RMF (0.4 T, 7.5 Hz, 2 h) significantly suppressed the metastasis of melanoma and survival time of the mice injected with B16-F19 cells (52). Actin cytoskeleton plays a major role in the process of driving cellular protrusions, such as lamellipodia and filopodia, at the leading edge of the cell, which is necessary for cell migration (96). In the absence of the geomagnetic field, also known as hypomagnetic field environment, the SH-SY5Y neuroblastoma cell adhesion and migration ability were diminished. Geomagnetic field shielding decreased the irregularity and eccentricity of the cell shape; cells maintain a weakened adhesive morphology, thicker, smaller, and rounder, which may be associated with its negative regulation of actin assembly (97).

### Differentiation

Moreover, the growth rate of tumors is closely related to the degree of tumor differentiation, which is an important reference index in cancer diagnosis and treatment. The LF-MF (5 mT, 50 Hz) was proved to cause an increase in 20% differentiation of hemin-induced K562 cells with a daily exposure of 1 h for 4 days (30). Another study found that the LF-MF (2 mT, 50 Hz, 96 h) exposure decreased the cellular proliferation potential and contributed to the ATRA-treated acute promyelocytic leukemia NB4 cell differentiation that varies with dose, where ROS and extracellular signal-regulated kinase (ERK) signaling pathways may be involved (31). These data suggested that MFs play promising roles as an assistant therapy in combination with other drugs to induce differentiation of leukemia cells. However, only a few studies have focused on the effects of MFs on the differentiation of cancer cells, and the mechanism involved might need a more detailed research.

## MFs in Combination Therapies

### In Combination With Chemotherapy and Other Therapies

**Figure 2:**
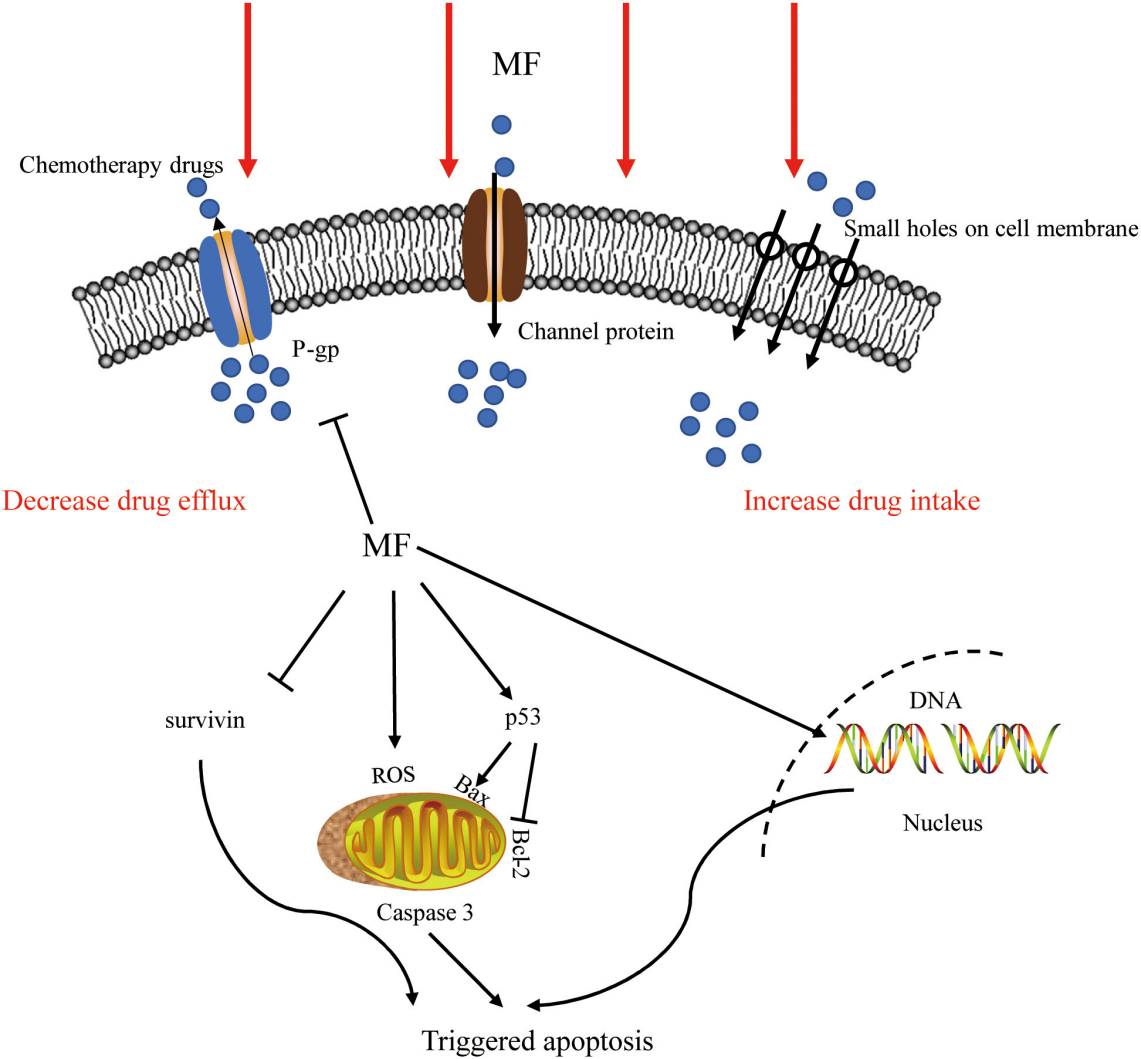
The effect of MF in combination therapy. The MFs not only increased the intake by producing small holes on the surface of the cell membrane and decreased the efflux of chemotherapeutic drugs by inhibiting ABC transporters but also affected ROS generation, DNA integrity, and apoptosis-related pathways to trigger apoptosis. P-gp, P-glycoprotein; MF, magnetic field; ROS, reactive oxygen species.

Chemotherapy always meets with increased toxicity and side effects caused by high dosage and drug resistance triggered by prolonged treatment, while combination therapy has obvious advantages by avoiding these. The co-treatment of SMF and cisplatin (10 ug/ml) for 12 h substantially suppressed the growth of K562 cells and augmented the chemosensitivity to cisplatin. This effect was correlated with the enhanced level of DNA damage and the arrest of the S-phase (27). Exposure to SMF with the intensity of 10 mT for 48 h led to a marked decrease in the viability percentage of cisplatin-treated HeLa cells through ROS accumulation (72). Appropriate SMF therapy increased the sensitivity of ovarian cancer cells, such as A2780 and A2780-CP, to cisplatin depending upon dose and exposure time, *via* producing small holes and large verrucous structures on the surface of the cell membrane (27, 98). The expression of P-glycoprotein is associated with multidrug resistance (MDR) in cancer cells, which is one of the main mechanisms of drug resistance in cancer cells (99). A combination with the SMF (8.8 m T, 12 h) decreased the expression of P-glycoprotein (P-gp) in K562 cancer cells, while adriamycin itself induced an increase (28). PMF (2 mT, 75 Hz, 1 h/day) coupled with temozolomide could slow down the proliferation of chemo- and radio-resistant T98G glioblastoma cell line by epigenetically affecting the regulation of oncogenes and tumor suppressors (100). The LF-MF (10 mT, 100 Hz, 144 h) promoted the sensitization of human glioblastomata, namely U87 and T98G, to temozolomide, which led to an increased apoptosis rate, with the evidence of increasing the expression of p53 and Bax and decreasing the expression of Bcl-2 and cyclin D1 (54). Capsaicin is the major pungent ingredient of the hot chili peppers, which could bind to distinct cell surface receptors including transient receptor potential vanilloid 1 (TRPV1) ion channel to exert anti-tumor function. An increased apoptosis rate was realized through the mitochondria-dependent apoptosis pathway, and the conformational change of TRPV1 triggered by the SMF (0.5 T, 72 h) might be the reason for this enhancement effect (101). Pre-exposure to 50 Hz LF-MF for 12 h and treatement with 5-fluorouracil (5-FU) for 24 h significantly inhibited the proliferation of MCF7 cells. This phenomenon was explained by increased DNA synthesis and upregulated cyclin E and cyclin D1 by the the MF to accumulate cancer cells at the S phase, which was more sensitive to 5-FU (102). The MF also showed a potential to retard tumor growth, elevate survival improvement, and reduce side effects when combined with radiotherapy and bacteriolytic therapy (43, 103). Therefore, the results of these studies support the fact that the MFs can be used as an adjunctive treatment to enhance the effects of chemotherapeutic drugs by increasing the DNA damage, cell apoptosis, and arresting the cell cycle, as summarized in Figure 2.

### Acting as a Drug Delivery System

**Figure 3:**
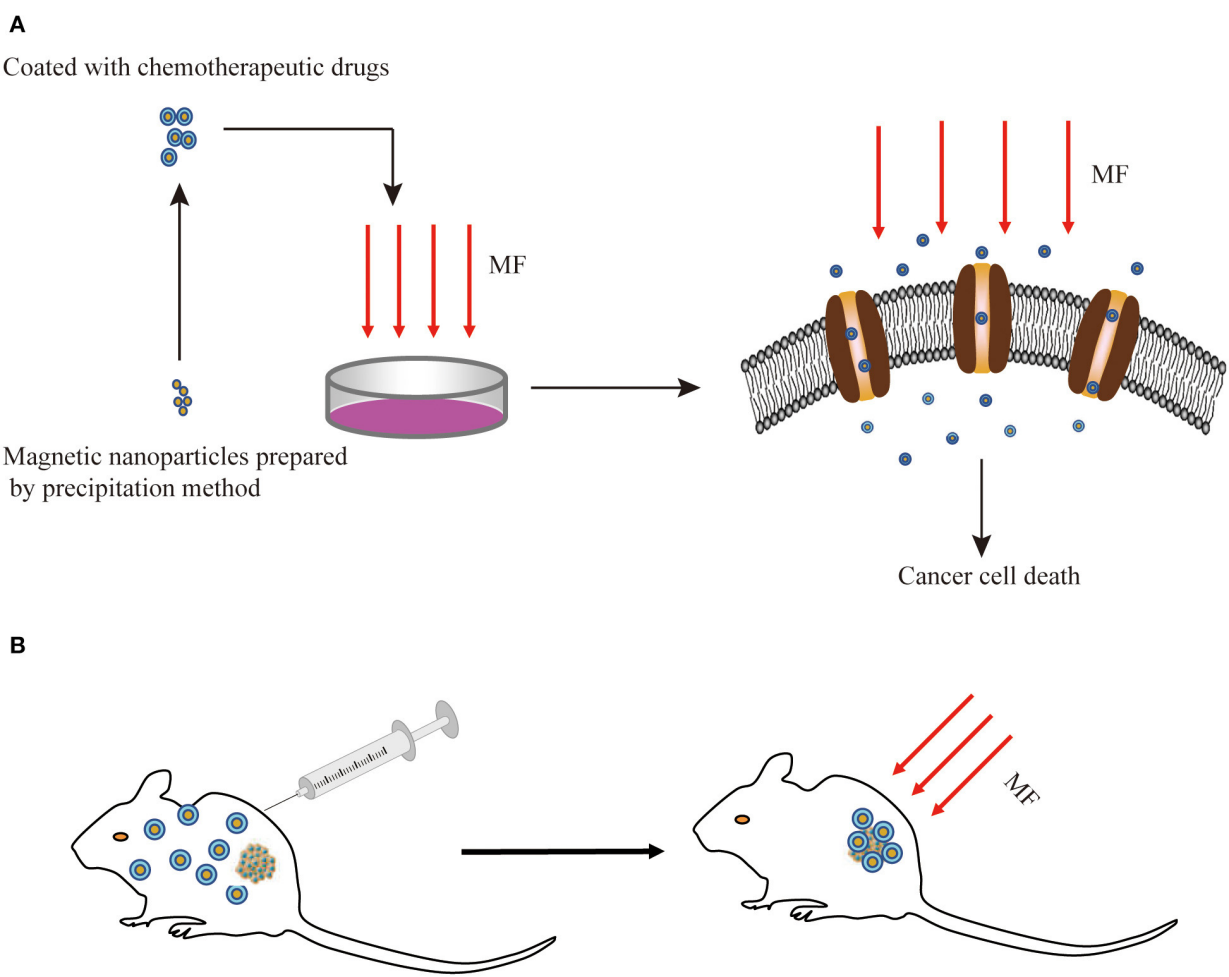
Schematic diagram of the combination of MFs with MNPs for cancer treatment *in vitro* **(A)** and *in vivo* **(B)**. MF, magnetic field; MNPs, magnetic nanoparticles.

Drug delivery systems (DDSs) were developed for targeting active biomolecules at the specific site of infection when treating patients with cancer, to improve the selectivity of the action sites of drugs, eliminate the side effects, and improve treatment efficiency. The MF targeting systems are always applied in combination with magnetic materials and anticancer drugs. Under the function of 100 Hz, 0.7 mT AMF, folic acid-modified magnetic nanoparticles (FA-MNPs) and alpha fetal protein monoclonal antibody-loaded MNPs (ATP-loaded MNPs) selectively induced the apoptosis of cancer cells and elevated the cellular iron uptake in a dose-dependent manner but had slight toxic effects on healthy cells (104, 105). The growth-inhibitory effects induced by SMFs and RMFs were enhanced by pretreating the cells with MNPs, while regulating the type and parameters of MFs could affect anti-tumor effects (106). The SMF along with low-intensity pulsed ultrasound (LIPUS) plus methotrexate (MT) prevented the growth of cancer cells better than bare drugs and single DDS, without any inhibition on the healthy cells (107). The detailed *in vitro* experiment results were subsequently validated *via in vivo* experiments, and the LIPUS+SMF DDS therapy improved at least 40% of the treatment efficacy, therapy reducing the natural activities of the cancer cells by changing the permeability, the potential of the cell membrane, and ROS generation (108). These results indicate that the MF could act as a DDS to target solid tumors in combination with MNPs to inhibit proliferation (Figure 3).

## Conclusion and Future Perspective

Numerous studies have shown that a wide range of types of MFs could affect the tumor cells at different degrees, while the dominant effects were associated with thermal or non-thermal mechanisms. The focus of this review is non-thermal effects, which were produced directly by the applied MFs themselves, instead of being produced indirectly as a result of heating. We summarized the performance, namely inhibiting cancer cell proliferation and inducing cell death in *in vitro* and *in vivo* models, of SMFs and LF-MFs in anti-tumor treatments. Also, co-treating with chemotherapy would achieve better therapeutic effects; meanwhile, the MF could serve as a DDS, targeting MNPs to the tumor, and the side effects are within the controllable range. Although various potential mechanisms of MFs against different cancer cell lines have been reported and discussed, few studies were performed on *in vivo* models. At present, most of these studies are confined to *in vitro* studies. Also, relevant clinical trials to test the safety and efficacy of MFs are not available. The limitations in these clinical studies might be due to their controversial roles in *in vitro* and *in vivo* studies, which are affected by some experimental variables such as the frequencies, intensities, or exposure duration of the MFs. Before clinical applications, there is still a demand for systematically exploring. Future studies should aim at finding optimum parameters at which these types of MFs will be most effective. Epidemiological studies have suggested that MFs at 50/60 Hz were also related to the development of depressive state anxiety, metabolic disturbance, poor sleep quality, and locomotor activity. However, according to ICNIRP, there is no sufficient scientific evidence for the association between MF exposure and these effects. Therefore, the most effective MF therapy should be tested further to guarantee its possible investigation in human. As for the MF devices, in consideration of the increasingly available clinical applications, the expectations should be portable and affordable. Future studies are expected to further determine the potential of the MF therapy in oncology.

## Author Contributions

TL and AX participated in the selection of papers and contributed to the writing of the paper. AX and QW collected, disposed, and analyzed the data. TL and XL helped to modify this manuscript. All authors contributed to the article and approved the submitted version.

## Conflict of Interest

The authors declare that the research was conducted in the absence of any commercial or financial relationships that could be construed as a potential conflict of interest.
